# Comparison Between Tracheal Wash and Bronchoalveolar Lavage Cytology for the Assessment of Airway Inflammation in Racehorses Affected by Exercise-Induced Pulmonary Hemorrhage

**DOI:** 10.3390/ani15172609

**Published:** 2025-09-05

**Authors:** Chiara Bozzola, Giulia Sala, Giovanni Stancari, Francesco Ferrucci, Enrica Zucca

**Affiliations:** 1 Department of Veterinary Medicine and Animal Science, Università degli Studi di Milano, 26900 Lodi, Italy; chiara.bozzola@unimi.it (C.B.); giovanni.stancari@unimi.it (G.S.); francesco.ferrucci@unimi.it (F.F.); 2 Department of Veterinary Sciences, Università di Pisa, 56122 San Piero a Grado, Italy; giulia.sala@unipi.it

**Keywords:** BAL, EIPH, lung inflammation, poor performance, pulmonary hemorrhage, TW

## Abstract

Exercise-Induced Pulmonary Hemorrhage (EIPH) results from alveolar capillary stress failure after strenuous exercise, leading to bleeding and pulmonary inflammation. Bronchoalveolar lavage fluid (BALF) cytology is considered the gold standard for the assessment of airway inflammation; however, it is often considered more invasive compared to tracheal wash (TW). It has been demonstrated that TW is a reliable diagnostic tool that may substitute BALF to diagnose and grade the severity of EIPH in racehorses; however, no information about airway inflammation in EIPH-affected horses has been reported. This retrospective study aimed to verify the agreement between TW and BALF in assessing lower airway inflammation in racehorses with EIPH. Cytological data from 123 poorly performing racehorses regarding neutrophils, eosinophils, and mast cells percentages were reviewed. Non-parametric statistical tests were used to assess the difference and the correlation between TW and BALF, and the distribution of inflammatory patterns in BALF. A strong correlation between TW and BALF was found for neutrophils (ρ = 0.817, *p* < 0.001) and eosinophils percentage (ρ = 0.806, *p* < 0.001); while a moderate correlation was found for mast cells percentage (ρ = 0.584, *p* < 0.001). The eosinophilic-mastocytic inflammation was the prevalent pattern observed in our population. Based on our findings, TW cannot be considered an alternative to BALF cytology for fully characterizing lower airway inflammation in racehorses with EIPH.

## Simple Summary

Exercise-induced pulmonary hemorrhage is a frequent respiratory disease in Standardbred and Thoroughbred racehorses performing high-level exercise. This condition has significant economic and welfare consequences in the racing industry, as it is frequently associated with reduced athletic performance and shortened career duration. Accurate diagnosis and monitoring of exercise-induced pulmonary hemorrhage are therefore crucial for both horse health and management decisions. Bronchoalveolar lavage fluid cytology is widely considered the gold standard for the diagnosis of lower airway inflammation. Since tracheal wash is perceived as less invasive than bronchoalveolar lavage fluid collection by trainers and owners, this retrospective study aimed to compare tracheal wash and bronchoalveolar lavage fluid cytology in the assessment of airway inflammation. Moreover, it aimed to assess the prevalence of different inflammatory cell types in 123 poorly performing racehorses affected by exercise-induced pulmonary hemorrhage. Our findings suggest that tracheal wash cannot be considered a reliable alternative to bronchoalveolar lavage fluid cytology for a comprehensive characterization of lower airway inflammation in racehorses affected by exercise-induced pulmonary hemorrhage. Therefore, the reliance on tracheal wash alone could lead to incomplete or misleading clinical assessments of airway inflammation in exercise-induced pulmonary hemorrhage-affected horses.

## 1. Introduction

Exercise-induced pulmonary hemorrhage (EIPH) is a common respiratory condition, defined as bleeding into the lung after strenuous exercise [1,2,3]. Epidemiological data suggest that EIPH is highly prevalent among racehorses, with endoscopic evidence of bleeding detected in most Thoroughbreds following competition [1,2,3].

The pathophysiology of EIPH is considered multifactorial, with hemodynamic, anatomical, and inflammatory mechanisms acting in concert. Exercise-induced pulmonary hemorrhage has been suggested to be due to alveolar capillary stress failure induced by the high pulmonary intra-vascular pressures achieved during intense exertion [4]. In strenuously exercising horses, transmural alveolar capillary pressure has been reported to be 25 mmHg, but this pressure, although very high, may not be sufficient to result in rupture of equine pulmonary capillaries. Therefore, it has been suggested that other factors beyond pulmonary arterial hypertension contribute to EIPH in horses [4]. Some histological studies identified extensive remodeling of small pulmonary veins, predominantly in the caudo-dorsal region of EIPH-affected lungs [4]. Blood flow is preferentially distributed to the caudo-dorsal lung regions in horses, at rest and especially during exercise, probably because of vascular anatomy and low regional vascular resistance [4]. Although pulmonary vascular pressure in the caudo-dorsal lung cannot be directly measured in exercising horses, it is reasonable to think that intravascular and transmural pressures of small veins are greatest in regions with low upstream vascular resistance and, therefore, high flow. Since remodeling is stimulated in veins exposed to higher pressures, it is mandatory to point out that racehorses are subjected to great pressure changes in caudo-dorsal lung regions during high exertion [4,5]. Therefore, pulmonary hypertension may cause pulmonary vessel wall remodeling, thickening, and reduction of venous lumen, and consequently local capillary hypertension and rupture, which may potentially lead to inflammation and angiogenesis, bleeding, hemosiderin accumulation, and lung fibrosis [5]. Consequently, the accumulation of blood within alveolar and interstitial tissues during pulmonary hemorrhage may worsen the inflammatory response, contributing to the development of repeated episodes of EIPH during strenuous exercise [5]. Moreover, given that recurrent hemorrhage can lead to hemosiderin deposition, oxidative stress, and progressive tissue remodeling, distinguishing whether inflammation reflects primary airway disease (such as mild-to-moderate equine asthma) or secondary responses to vascular injury is essential [1,4,5].

In recent years, the association between EIPH and airway inflammation has been investigated, showing controversial results. Experimental studies have shown that autologous blood inoculated into the airways increases the percentage of alveolar macrophages and hemosiderophages. The presence of blood induces airway inflammation in horses, with a notable rise in neutrophil percentage within the first 24 h post-inoculation [6,7]. A large-scale study involving 148 Thoroughbred racehorses identified an association between signs of airway inflammation and EIPH, particularly an increase in neutrophil count was found by tracheal wash (TW) cytology [8]. However, other studies showed no correlation between EIPH and airway inflammation [9], tracheal mucus score [9], or the presence of hemosiderophages in TW and clinical signs such as coughing [10].

Assessment of airway inflammation and EIPH in horses is typically performed through cytological analysis of tracheal wash (TW) and bronchoalveolar lavage fluid (BALF) samples. Although BALF provides more representative samples from the lower respiratory tract, it is often considered less practical and more invasive compared to TW [11,12]. Several studies have compared the two techniques to evaluate their agreement in characterizing airway inflammation and in diagnosing different respiratory diseases in horses (such as equine asthma and EIPH) [13,14,15,16,17,18].

To the best of the author’s knowledge, there are no studies that compare TW and BALF cytology to assess airway inflammation in racehorses affected by EIPH. Therefore, the present study aimed to investigate the agreement between TW and BALF in the assessment of neutrophils, eosinophils, and mast cell count in Standardbred and Thoroughbred racehorses affected by EIPH. Additionally, it aimed to evaluate the prevalence of different types of airway inflammation (neutrophilic, eosinophilic-mastocytic, or mixed inflammation) on BALF cytology.

The importance of this investigation lies in the fact that EIPH not only compromises the respiratory health of affected horses but also has a direct economic impact on the equine industry. Poor performance due to recurrent bleeding episodes often leads to reduced racing potential, earlier retirement, and significant financial losses for owners and trainers. Furthermore, EIPH represents a welfare issue, as repeated bleeding events can compromise the long-term pulmonary function of the animal, with possible progression to chronic lung remodeling and fibrosis. Therefore, understanding the interplay between airway inflammation and EIPH is critical for improving diagnostic strategies, therapeutic interventions, and preventive measures aimed at protecting equine athletes.

## 2. Materials and Methods

### 2.1. Horses

The horses included in the present study were retrospectively selected among client-owned patients referred to the Equine Veterinary Hospital of the University of Milan (Italy) for poor performance evaluation, between 2001 and 2024. Inclusion criteria were the presence of TW and BALF cytology collected at the same instance in racehorses (Standardbred and Thoroughbred), and the presence of hemosiderophages on BALF cytology. Due to the absence of consensus on a cut-off for the percentage of hemosiderophages indicating a positive diagnosis of EIPH, we chose to include only horses with at least hemosiderophages ≥ 10% on BALF cytology [19]. Moreover, to minimize the risk of bias related to pharmacological modulation of inflammatory cell counts, horses treated with corticosteroids, bronchodilators, or other medications with potential anti-inflammatory effects within two weeks prior to examination were excluded.

All the medical procedures were performed for diagnostic purposes in clinical patients, and informed consent was signed by all the owners for the use of clinical data for research aims. As this was a retrospective study, all data were processed following the international legislation regarding ethical research on animals, without requiring a submission of the protocol to the Institutional Animal Care and Use Committee.

### 2.2. Diagnostic Protocol

Horses admitted for poor performance evaluation underwent a complete diagnostic protocol, including resting examination, standardized incremental treadmill test for the evaluation of lactate curve, high-speed treadmill endoscopy, and post-exercise tracheobronchoscopy (30 min after the end of the high-speed treadmill test). At least 24 h later, tracheal wash and bronchoalveolar lavage fluid were collected as previously described [18,20,21,22]. Briefly, the horses were restrained in the stocks, sedated with detomidine hydrochloride (0.01 mg/kg IV), and a nose twitch was applied. A 13 mm diameter 170 cm long flexible video endoscope (EC-530WL-P, Fujifilm, Tokyo, Japan) was passed through one of the nostrils, into the pharynx, and then inserted into the trachea. To collect tracheal wash, 60 mL of sterile 0.9% saline solution was flushed into the intrathoracic portion of the trachea and re-aspirated. To collect bronchoalveolar lavage fluid, a sterile catheter was put into the service canal of the endoscope, and 60 mL of a 0.5% lidocaine hydrochloride solution was sprayed at the level of the tracheal bifurcation to reduce the coughing reflex. The endoscope was then passed through the right main bronchus until it was wedged, and here, a total of 300 mL of sterile 0.9% saline solution was instilled using the sterile catheter [18]. The fluid was immediately re-aspirated. Samples were stored in sterile ethylenediaminetetraacetic acid (EDTA) tubes for cytology. Slide preparation was performed immediately or at least 1 h after sample collection. A few drops of TW and BALF samples were cytocentrifuged (Rotofix 32, Hettich Cyto System, Tuttlingen, Germany) for 5 min at 500 rpm. The slides were air-dried, stained with May–Grünwald Giemsa (MGG) and Perl’s Prussian Blue (PPB), and then observed under a light microscope at 400X and 1000X for 400-cell differential counting. Then the percentage of hemosiderophages of the total macrophages was calculated, and the hemosiderin was scored from 0 to 4 based on the blue coloration of the macrophages’ cytoplasm [23]. A score 0 was defined as the absence of blue coloration in the cytoplasm, a score 1 was defined as faint blue staining in the cytoplasm, a score 2 was recorded when a dense blue color in a minor portion of the cytoplasm or medium color intensity throughout the cell was evident, a score 3 was assigned when a deep blue staining in most of the cytoplasm was found, and a score 4 was described as cell filled with hemosiderin, dark blue throughout the cytoplasm [23]. Sample preparation and interpretation of all cytological specimens were carried out by the same operator with specific training in equine respiratory cytology.

### 2.3. Retrospective Data Assessment

Among horses affected by EIPH (percentage of hemosiderophages ≥ 10% on BALF cytology), the cytology records (TW and BALF) were reviewed for the percentage of neutrophils, eosinophils, and mast cells.

Based on the type of airway inflammation detected on BALF cytology (neutrophilic inflammation, eosinophilic-mastocytic inflammation, mixed inflammation, and no inflammation), the horses were divided into 4 groups. The first group had neutrophils ≥ 5% (neutrophilic inflammation); the second group had eosinophils ≥ 0.5% and/or mast cells ≥ 2% (eosinophilic-mastocytic inflammation); the third group had neutrophils ≥ 5% and eosinophils ≥ 0.5% or mast cells ≥ 2%, or all three (mixed-inflammation); the fourth group had neutrophils < 5%, eosinophils < 0.5%, and mast cells < 2% (no inflammation) [24]. Furthermore, records of bronchoalveolar lavage fluid cytology regarding the hemosiderin content in the macrophages’ cytoplasm were reviewed.

### 2.4. Statistical Analysis

All data were collected in a spreadsheet (Microsoft Excel, Office package), and the statistical analysis was performed using IBM SPSS Statistics, version 29.0 (IBM Corp., Armonk, NY, USA). The Shapiro–Wilk test was used to assess the normality of distribution for all continuous variables. Since the data did not follow a normal distribution, results were expressed as median, 25th percentile, and 75th percentile. Categorical variables were reported as frequencies and percentages. A *p*-value < 0.05 was considered statistically significant.

Differences between tracheal wash and bronchoalveolar lavage fluid cytology in neutrophils, eosinophils, and mast cell counts were assessed separately using the Mann–Whitney U test. The correlation between TW and BALF cytology for the same inflammatory cells was evaluated using Spearman’s rank correlation coefficient (rho, ρ). The strength of correlation was interpreted as follows: values of ρ < 0.10 indicated negligible correlation, values between 0.10 and 0.39 indicated weak correlation, values between 0.40 and 0.69 indicated moderate correlation, values between 0.70 and 0.89 indicated strong correlation, and values between 0.90 and 1.00 indicated very strong correlation [25]. The distribution of inflammatory patterns in BALF cytology was evaluated using the chi-square test. Finally, the Kruskal–Wallis test was used to assess differences in the percentage of neutrophils, eosinophils, and mast cells among the different hemosiderin scores (from 0 to 4), based on the hemosiderin content in the macrophages’ cytoplasm on bronchoalveolar lavage fluid cytology.

## 3. Results

### 3.1. Horses

Of a total of 475 horses, 123 met the inclusion criteria, of which 15 (12.2%) were Thoroughbred, while 108 (87.8%) were Standardbred. A total of 42 (34.1%) were female, 68 (55.3%) were male, and 13 (10.6%) were geldings. The median age was 3 years old (3–5 years old), whereas the median body weight was 452 kg (435–479 kg).

### 3.2. Statistical Results

Median, 25th percentile, and 75th percentile for the percentage of neutrophils, eosinophils, and mast cells are reported in Table 1.

No significant differences (*p* > 0.05) were found in the assessment of neutrophil and eosinophil percentage between tracheal wash and bronchoalveolar lavage fluid cytology; while a statistically significant difference (*p* < 0.001) was found for mast cell count, with a higher proportion of these cells in BALF cytology. A strong correlation between TW and BALF cytology was found for neutrophil (ρ = 0.817, *p* < 0.001) and eosinophil percentage (ρ = 0.806, *p* < 0.001); a moderate correlation was found for mast cell percentage (ρ = 0.584, *p* < 0.001).

Based on BALF cytology, 3/123 (2.5%) horses had no airway inflammation, 80/123 (65.0%) horses had an eosinophilic-mastocytic inflammation, 6/123 (4.9%) had a neutrophilic inflammation, and 34/123 (27.6%) had a mixed inflammation.

Regarding the hemosiderin scores on BALF cytology, 5/123 (4.1%) horses had a score of 1, 60/123 (48.8%) horses had a score of 2, 52/123 (42.3%) horses had a score of 3, and 6/123 (4.8%) horses had a score of 4. No significant differences were found in the assessment of neutrophil (*p* = 0.115), eosinophil (*p* = 0.557), and mast cell (*p* = 0.419) percentage among the different hemosiderin scores.

## 4. Discussion

While the pathophysiology of EIPH has been extensively studied, the relative importance of inflammation as a causal factor versus a secondary consequence remains unresolved [5,8]. In this context, cytological examination of respiratory tract secretions has been a cornerstone in equine medicine, allowing characterization of inflammatory phenotypes that may otherwise remain undetected by endoscopy alone.

Tracheal wash and bronchoalveolar lavage fluid are the two most widely employed sampling techniques in the diagnostic evaluation of equine lower airway disease. Tracheal wash is easier to obtain, minimally invasive, and more suitable for large-scale or field-based assessments, but its diagnostic accuracy has long been debated due to its limited representation of distal lung pathology [11,12]. Bronchoalveolar lavage fluid samples, in contrast, provide a more reliable reflection of cytological processes within the lower respiratory tract and are widely regarded as the gold standard in the assessment of airway inflammation. However, its more invasive nature, need for sedation, and technical limitations restrict its applicability in routine practice [11,12]. For these reasons, the degree of concordance between tracheal wash and bronchoalveolar lavage fluid cytology remains a crucial area of investigation in horses with EIPH, as it may determine which method is most appropriate for both clinical and research settings.

In the present study, a strong correlation between tracheal wash and bronchoalveolar lavage fluid cytology was found for the eosinophil count. This is in line with other studies reported in the literature, both in clinically normal and poorly performing horses [15,16,26], where no significant differences between tracheal wash and bronchoalveolar lavage fluid cytology were found for this inflammatory cell line.

Our findings showed a strong correlation between TW and BALF neutrophil count as well. Rossi et al. (2018), comparing tracheal wash and bronchoalveolar lavage fluid cytology in a population of 154 horses with and without signs of respiratory diseases, found that TW was more sensitive and specific in detecting lower airway diseases and reported a substantial agreement with BALF in neutrophil percentage [13]. However, another study reported a high level of disagreement (37%) between tracheal wash and bronchoalveolar lavage fluid cytology in young poorly performing Australian racehorses, judging TW to be more sensitive in detecting airway neutrophilia [14]. However, it is important to highlight that the results of this second paper may be influenced by the collection of tracheal wash and bronchoalveolar lavage fluid samples within 1–2 h after high-speed treadmill exercise. In fact, it has been demonstrated that recent exercise increases neutrophil count in tracheal wash fluid [27].

As already reported in horses affected by lung diseases [15,16], the mast cell count was different between tracheal wash and bronchoalveolar lavage fluid cytology, also in our study, with a higher percentage in bronchoalveolar lavage fluid samples. Although the reason for this difference is not known yet, studies on other species found that mast cells are highly heterogeneous in size, granule content, level of degranulation, and staining patterns, depending on their localization in the airways (trachea or lung) [28,29]. According to these studies, it has been suggested that several mast cell subpopulations could be detected within the equine respiratory mucosa as well, each potentially exhibiting unique response profiles and different local migration to and from the airways [15].

Airway inflammation was highly prevalent in our population of racehorses affected by EIPH, with only 2.5% of them showing no evidence of inflammation on bronchoalveolar lavage fluid cytology. Lower airway inflammation has long been proposed as a contributing factor in the development or progression of EIPH in horses [30]. Inflammation may influence the onset or progression of exercise-induced pulmonary hemorrhage through two main mechanisms. Firstly, inflammation and bronchoconstriction in the lower airways can lead to partial obstruction within the airways, leading to more negative intra-alveolar pressures that may induce the development of pulmonary hemorrhage [8,30,31]. Secondly, the accumulation of blood within alveolar and interstitial tissues during pulmonary hemorrhage may itself provoke an inflammatory response, potentially contributing to the persistence or worsening of EIPH already present [5]. These pathophysiological processes may explain why many horses develop chronic, recurrent episodes of EIPH despite supportive management. Future studies should therefore aim to investigate whether targeted anti-inflammatory treatments could reduce the frequency or severity of hemorrhage episodes. Although it remains unclear whether the inflammation precedes EIPH or results from it, our findings are consistent with previous experimental [6,7] and observational [8,31] research, in which an association between EIPH and airway inflammation was found.

The predominant inflammatory pattern observed in our population was eosinophilic-mastocytic inflammation (65.0%), followed by mixed (27.6%) and neutrophilic (4.9%) inflammation. Based on the ACVIM Consensus Statement of Airway Inflammatory Disease (IAD) of Horses [32], an increase in eosinophil, mast cell, and/or neutrophil percentages in BALF cytology is typically observed in young, poorly performing racehorses in training. IAD is now called mild-moderate equine asthma (MEA) and can have a prevalence higher than 80% in young racehorses [24,32,33,34]. Our results are consistent with previous studies in the literature, in which an association between MEA and EIPH has been reported in horses [8,35].

The strong correlation between TW and BALF neutrophil and eosinophil counts might suggest that both sampling techniques may be useful for routine screening of inflammatory airway conditions in the clinical setting. However, the discrepancy in mast cell detection is particularly relevant, as mast cells are increasingly recognized as key modulators of airway reactivity and inflammation. Their role in bronchoconstriction, vascular permeability, and tissue remodeling highlights the necessity of their accurate quantification [36]. The underestimation of mast cells in TW cytology could therefore lead to misclassification of the inflammatory phenotype, with potential implications for the selection of appropriate therapeutic strategies. Given that the predominant inflammatory phenotype observed in our study was eosinophilic–mastocytic, our findings suggest that TW is not a reliable method for evaluating airway inflammation in racehorses affected by exercise-induced pulmonary hemorrhage. Previous research showed a poor correlation between TW and BALF in the cytological evaluation of lower airway inflammation, emphasizing that TW cannot be considered a suitable alternative to BALF cytology when a definitive diagnosis or accurate characterization of lower airway inflammation is required [14,15,16,32,37]. Accordingly, our results support the notion that bronchoalveolar lavage fluid cytology remains the gold standard for evaluating lower airway inflammation in racehorses with EIPH. Conversely, TW is considered a reliable tool for the assessment of EIPH in poorly performing racehorses [18].

No differences in the percentage of neutrophils, eosinophils, and mast cells among the different hemosiderin scores were found in the present study. However, this result might be because scores 2 and 3 were the most represented in our population, with 60 and 52 horses included, respectively.

Currently, a standardized cutoff for hemosiderophage percentage to distinguish horses with and without EIPH is lacking in the literature, making the comparison between studies very challenging. Our study aimed to assess cytological differences in inflammatory cell populations between tracheal wash and bronchoalveolar lavage fluid cytology in horses with EIPH, regardless of the absolute amount of hemosiderophages in BALF cytology. For this reason, according to a classification reported in the literature [8,19], we chose to include horses with at least ≥10% of hemosiderophages in BALF cytology.

Finally, the potential influence of breed and age on the prevalence of different inflammatory profiles was not investigated in the present study. However, it should be noted that Standardbred horses made up the majority of our study population, and the age distribution was relatively homogeneous (median age 3 years old).

## 5. Conclusions

Exercise-induced pulmonary hemorrhage represents a multifactorial condition in racehorses, with both mechanical and inflammatory components contributing to its onset and progression. Although bleeding into the lower airways is a defining feature, the interplay between airway inflammation, vascular stress, and repetitive high-intensity exercise remains incompletely understood. Understanding the patterns of inflammatory cell infiltration in affected horses is essential for clarifying disease mechanisms and optimizing management strategies. While multiple sampling techniques exist to assess airway inflammation, the relative accuracy and clinical utility of these methods, particularly in relation to specific cell populations, continue to be debated.

Our findings highlight that while tracheal wash and bronchoalveolar lavage fluid cytology may align for neutrophil and eosinophil counts, significant discrepancies in mast cell percentage limit the reliability of TW, and BALF cytology remains the gold standard for fully characterizing airway inflammation in racehorses with exercise-induced pulmonary hemorrhage. The clinical implication of this work is that veterinarians should be cautious when relying on tracheal wash cytology alone in the diagnostic evaluation of poorly performing racehorses. In fact, while TW may provide an accessible first-line tool for identifying racehorses suffering from exercise-induced pulmonary hemorrhage, BALF should be considered mandatory in cases where accurate characterization of lower airway inflammation is required.

Moreover, the high prevalence of eosinophilic-mastocytic inflammation in EIPH-affected horses highlights the need for further research into the role of these cell populations in equine respiratory pathology. Ultimately, a better understanding of the interplay between bleeding, inflammation, and airway remodeling will not only improve the welfare and athletic performance of racehorses but also help refine preventive and therapeutic strategies in equine sports medicine.

## Figures and Tables

**Table 1:** Median, 25th percentile, and 75th percentile for the percentage of hemosiderophages, simplified Total Hemosiderin Score, and percentage of neutrophils, eosinophils, and mast cells of all horses (n = 123).

	TW	BALF
Neutrophils (%)	15 (6.0–24.0)	13 (6.0–20.0)
Eosinophils (%)	1 (0.0–2.0)	1 (0.0–3.0)
Mast cells (%)	2 * (1.0–3.0)	4 * (3.0–6.0)

* The same subscriptions indicate a statistically significant difference (*p* < 0.05) between TW and BALF cytology. BALF, bronchoalveolar lavage fluid; TW, tracheal wash.

## Data Availability

Data presented in this study are available on request from the corresponding author.
